# The General Practitioner Prompt Study to Reduce Cardiovascular and Renal Complications in Patients With Type 2 Diabetes and Renal Complications: Protocol and Baseline Characteristics for a Cluster Randomized Controlled Trial

**DOI:** 10.2196/resprot.9588

**Published:** 2018-06-08

**Authors:** Andrew Willis, Winston Crasto, Laura Gray, Helen Dallosso, Ghazala Waheed, Geri Gray, Melanie J Davies, Kamlesh Khunti

**Affiliations:** ^1^ Diabetes Research Centre University of Leicester Leicester United Kingdom; ^2^ George Eliot Hospital NHS Trust Nuneaton United Kingdom; ^3^ Department of Health Sciences University of Leicester Leicester United Kingdom; ^4^ NIHR Biomedical Research Centre University of Leicester Leicester United Kingdom

**Keywords:** diabetes mellitus, type 2, primary health care

## Abstract

**Background:**

Adherence to evidence-based cardiovascular risk factor targets in patients with type 2 diabetes and microalbuminuria has shown long-term reduction in mortality and morbidity. Strategies to achieve such adherence have been delivered at individual patient level and are not cost-effective. Health care professional-level intervention has the potential to promote better adherence at lower cost.

**Objective:**

The aim of this study was to assess the effectiveness of a multifactorial technology-driven intervention comprising health care professional training, a software prompt installed on practice systems, clinician email support, and enhanced performance and feedback reporting.

**Methods:**

A cluster randomized trial will be performed where the primary outcome is the proportion of eligible patients meeting tight cardiovascular risk factor targets, including systolic and diastolic blood pressure (BP; BP<130/80 mm Hg) and total cholesterol (TC; TC<3.5 mmol/L) at 24 months. Secondary outcomes include proportion of patients with glycated hemoglobin (HbA_1c_) <58 mmol/mol (7.5%), change in medication prescribing, changes in microalbuminuria and renal function (estimated glomerular filtration rate, eGFR), incidence of major adverse CV events and mortality, and coding accuracy. Cost-effectiveness of the intervention will also be assessed.

**Results:**

Among 2721 eligible patients, mean age was 62.9 (SD 10.0) years, and duration of diabetes was 10.46 (SD 7.22) years. Mean HbA_1c_ was 59.3 (SD 17.4) mmol/mol; mean systolic and diastolic BP (mm Hg) were 134.3 (SD 14.6) and 76.1 (SD 9.5) mm Hg, respectively; and mean TC was 4.1 (SD 0.98) mmol/L. Overall, 131 out of 2721 (4.81%) patients achieved all 3 “tight” cardiovascular risk factor targets. Cardiovascular risk factor burden increased two-fold in those with eGFR<60 mL/min/1.73 m^2^ compared with those with eGFR≥60 mL/min/1.73 m^2^. Prevalence of microalbuminuria was 22.76%. In total, 1076 out of 2721 (39.54%) patients were coded for microalbuminuria or proteinuria on their primary care medical record.

**Conclusions:**

The general practitioner prompt study is the largest UK primary care-based, technology-driven, randomized controlled trial to support intensive intervention in high-risk group of multiethnic individuals with type 2 diabetes and microalbuminuria. This paper provides contemporary estimates for prevalent cardiovascular disease and adherence to evidence-based cardiovascular risk factor targets at baseline in a population with type 2 diabetes and microalbuminuria. The main trial results, including cost-effectiveness data, will be submitted for publication in 2018.

**Trial Registration:**

International Standard Randomized Controlled Trial Number ISRCTN14918517; http://www.isrctn.com/ISRCTN14918517 (Archived by WebCite at http://www.webcitation.org/6zqm53wNA)

**Registered Report Identifier:**

RR1-10.2196/9588

## Introduction

### Type 2 Diabetes and Microalbuminuria

Microalbuminuria (MA) in patients with type 2 diabetes (T2DM) is associated with a significantly increased risk of cardiovascular (CV) mortality and related morbidity [1]. Current evidence advocates targeted, tight, multiple risk factor control to reduce CV risk [2]. Recent audit data suggest that despite current guidance, over 80% of patients with MA and T2DM do not meet all treatment targets for blood pressure (BP), total cholesterol (TC), and glycated hemoglobin (HbA_1c_) [3].

### Evidence for Tighter Cardiovascular Risk Factor Control

Previous studies have demonstrated the effectiveness of patient-level interventions using tighter treatment targets [4,5] including group patient education [3,6], showing long-term beneficial microvascular and macrovascular benefits in addition to reduced mortality.

Strategies to improve quality of care in diabetes, including health care professional (HCP) education, providing financial incentives, professional reminders, and audit and feedback, have generally reported improvements in care, albeit with modest reductions in HbA_1c_ [7]. Due to differences in interventions, outcome measures, and study populations, it is difficult to compare data on effectiveness between clinician- and patient-focused interventions [8].

Although there is evidence to suggest that patient-level interventions to manage CV disease (CVD) are cost-effective [9], questions loom over the ability to implement them with limited resources available in primary care settings. Simple prompts integrated into existing information technology (IT) systems to identify patients during routine consultation, in combination with education for clinicians, may serve as an “aide-mémoire” and provide an opportunity to improve standards of care at low cost [10-12]. More specifically, it has been shown to improve adherence to medication in studies targeting a reduction in CVD risk in people with T2DM [13,14]. The provision of patient “reminders” and audit and feedback are facilitated through existing practice IT systems [15]. This highlights opportunities for intervention in patients at the time of clinical encounter [16] and is effective in improving HCP behavior to achieve patient risk factor targets [15].

### The General Practitioner-Prompt study

The general practitioner (GP) prompt study was designed to test the hypothesis that a multifaceted, multifactorial intervention in patients with T2DM and MA aimed at primary care HCPs aided with an electronic “Prompt” would result in a selective, intensive, and targeted intervention of CV risk factors in these high-risk individuals and an increase in the proportion meeting tight multiple CV risk factor targets [2].

## Methods

### Study Design

This study is a pragmatic cluster randomized controlled trial (RCT). Ethics approval was granted by the National Research Ethics Committee: North West Lancaster on March 16, 2015 (ref: 166517).

The rationale for randomizing at the cluster level was that the intervention was implemented across all eligible patients, and treatment decisions regarding individual patients remained the responsibility of HCPs at each practice [17,18].

The duration of this trial is 24 months. The installation of the prompt took place on November 1, 2015. Patients registered with control practices continued to receive usual care in line with current best practice guidelines [2].

This paper reports the trial design and baseline biomedical characteristics, including CV risk factor burden and medication management data in this study population.

### Setting

Eligible practices (using EMIS Web or SystmOne IT systems and a list size of >6000 patients) within the recruitment area of Leicester City and Leicestershire County were sent an invitation summarizing the study design and protocol. Staff members who expressed an interest were offered a meeting with a member of the study team to clarify any queries regarding the study. Informed consent and information governance approval was sought among eligible practices willing to participate and documented from a senior GP partner or practice manager and Caldecott Guardian. Practices were then randomized by a member of the Leicester Clinical Trial Unit (not involved with the study) to the intervention or control arm. A 1:1 randomization was stratified based on size of diabetes register (small practices <600 patients, large practices >600 patients).

### Patient Inclusion Criteria

Patient-level data were extracted for individuals aged between 17 and 76 years with a Read code for T2DM and MA or overt proteinuria on their clinical record, or individuals with T2DM and albumin-creatinine ratio (ACR)>2.5 in males and >3.5 in females on 2 consecutive occasions of >90 days and <180 days apart, having excluded a urinary tract infection [2].

### Patient Exclusion Criteria

Data were not extracted if patients fulfilling the inclusion criteria were pregnant, terminally ill, or excluded from the Pay for Performance—Quality and Outcomes Framework (QOF; whole domain diabetes) [19].

### Intervention

The multifactorial intervention uses an IT software prompt and care template to alert HCPs to eligible patients attending a routine consultation (Multimedia Appendices 1 and 2). The software prompt and care template was developed by an external software company, with refinement informed by feedback from HCPs during an initial focus group. The prompt and care template is triggered when patients with T2DM and MA as well as BP, TC, or HbA_1c_ above target attend a consultation. The HCP is alerted to risk factors that are above target and displays the patient’s last 12-month values for each risk factor. The prompt also allows the clinician to access an evidence-based treatment algorithm recommending specific therapies that can be followed to achieve tight-targeted risk factor control (Multimedia Appendix 3). If sufficient control cannot be achieved, a link to a study email address is available for HCPs to request further individualized advice. This advice is provided within 1 week via email by a study clinician. An existing patient education leaflet emphasizing the importance of treatment adherence will also be available for eligible patients. Practice staff attended training before the prompt was installed and are provided with ongoing support and feedback during the study period (Multimedia Appendix 4).

### Primary and Secondary Outcomes

The primary outcome is the proportion (%) of eligible patients meeting both of the following CV risk factor targets: BP<130/80 mm Hg and TC<3.5 mmol/L. These outcomes were selected as they are current recommended care processes and clinical outcomes of care for the management of individuals with T2DM and MA [20,2].

Secondary outcomes include the following: incidence of CV events and all-cause mortality, smoking status glycemic control assessed by HbA_1c_, progression in MA assessed by change in ACR, kidney function measured by change in estimated *glomerular filtration rate* (e*GFR*), changes in T2DM, BP, and cholesterol-lowering medication prescribing, including contraindications and adverse reactions. Data extracted relate to blood samples that are previously collected as part of routine care and analyzed in accordance with relevant regulations and standard operating procedures.

### Sample Size Considerations

Assuming 7.5% of patients with T2DM and MA meet enhanced targets for BP (<130/80 mm Hg) and TC (<3.5 mmol/L) in the standard care group with an intraclass correlation of .05, an average of 118 patients with MA per practice (ranging from 27 to 549), data are required from 18 practices (9 in each arm) to detect an increase to 18% or higher in the proportion meeting both enhanced targets in the intervention group, with 80% power at the 5% significance level. The inflation for unequal cluster size is based on a coefficient of variation of 1.11 [21]*.*

### Data Extraction

Primary outcome is measured at baseline, 12, and 24 months post randomization in control practices and every 3 months in intervention practices to allow reporting of audit and feedback data to this group of practices.

One line per patient anonymized data is extracted using a standardized morbidity information query and export syntax (MIQUEST) query [22] in line with local governance regulations [23,24]. Time frames for data extraction are shown in Table 1. Data extraction is carried out remotely using Away from My Desk (Away From My Desk Limited, United Kingdom) [25] software. Results are uploaded to a secure online database and transferred to the research team via encrypted National Health Service email systems.

### Analysis Plan

We are using a cluster randomized design with repeated measurements, and therefore, there is a high likelihood that biomedical characteristics may correlate within a cluster. We will perform linear and logistic multilevel regression analyses to study the effects of the intervention with cluster as the random effect, adjusted for the baseline value of the outcome both at practice and individual levels, using the missing indicator method for “missing” baseline data. Data will be analyzed as intention-to-treat, and differences in outcomes measures between the intervention and control groups will be calculated with 95% CI. It is also likely that not all individuals from the control group will have had regular appointments with their GP; hence, we will perform a sensitivity analysis to compare “attenders” at similar time points in the intervention versus control group.

For presentation of baseline data, we will use Pearson chi-square to analyze differences in proportions between intervention and control groups. Independent samples *t* tests will be used to analyze differences in continuous variables between study groups. Statistical analyses of the baseline data and all future analysis will be carried out using STATA version 14 [26].

### Modeling the Economic Costs of the Intervention

Decision-analytic modeling will be undertaken to estimate the long-term effectiveness of the intervention compared with usual care. The costs of setting up and providing the intervention are being collected. Ongoing costs will be combined with unit costs to produce the total cost of the intervention over the 24-month study period. Unit costs for health care resources will be calculated from local and national sources and standardized to current prices. Comparisons of the primary outcome measure (individuals achieving BP and TC clinical targets) between baseline and 24-month follow-up interventions will be used to estimate the costs and incremental cost-effectiveness ratios.

The average number of eligible patients per practice will be used to estimate costs at the practice and Clinical Commissioning Group levels. All costs will be for 2016. Salary costs will be taken from Curtis (2015, [27]), and the cost of laboratory tests will be provided by the Leicester Pathology Service. Time to undertake tasks will be modeled with uncertainty in the analysis.

## Results

**Figure 1:**
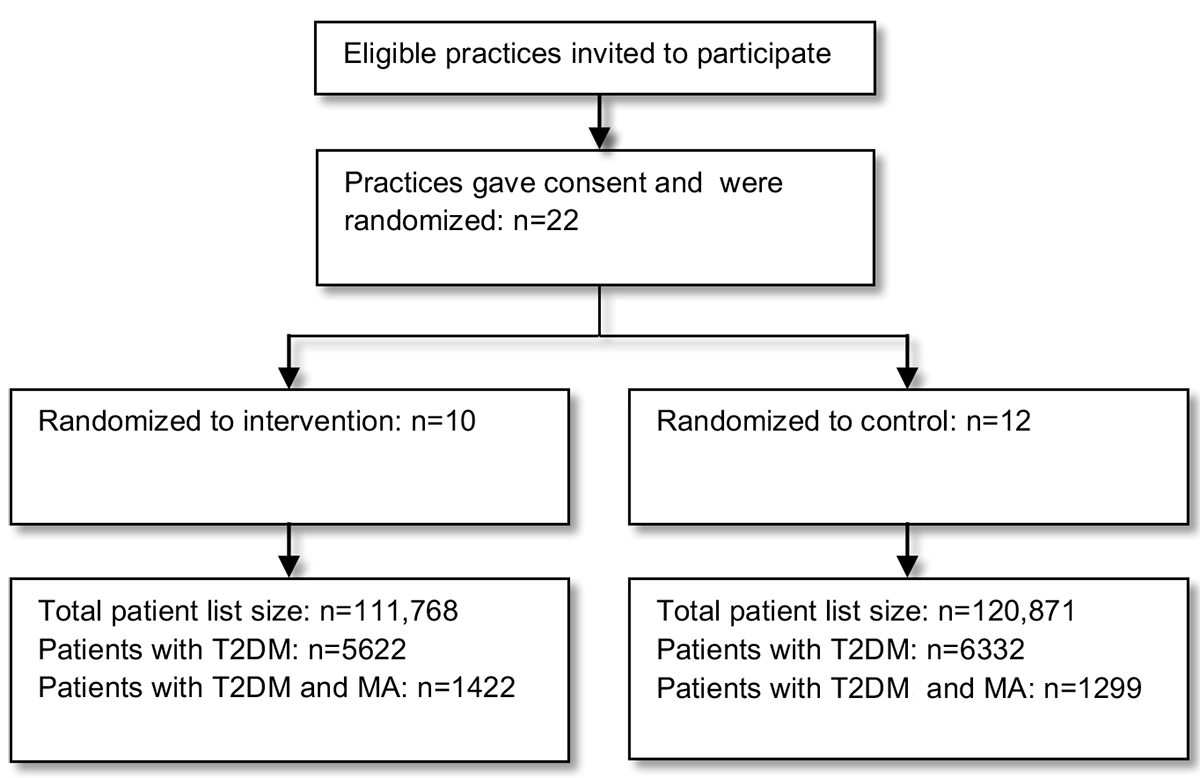
Recruitment flowchart. T2DM: type 2 diabetes; MA: microalbuminuria.

Baseline data have been extracted from 22 practices (12 controls and 10 interventions; Figure 1) with a reference date of October 30th, 2015. The total number of patients registered at participating practices is 232,639. There are 2721 patients with T2DM meeting the study MA criteria and eligible for the study. Of these 2721, 1067 patients (39.5%; 95% CI 37.7-41.4) had a code for MA or proteinuria in their electronic medical notes.

Biomedical characteristics, current risk factor control, medical history, and current drug prescriptions of the study population are shown in Tables 1-3. The mean age of patients is 62.9 (SD 10.0) years. There are no significant differences in number of male or female patients, and the study population, in keeping with local demographics, is predominantly of South Asian ethnicity (1136/1838 patients, 61.77%). The mean duration of T2DM is 10.5 (SD 7.2) years. Out of 2721 participants, 415 (15.25%) and 739 (27.16%) patients are current smokers and ex-smokers, respectively. Out of 2721 patients, 536 (19.70%) have chronic kidney disease stage 3.

The mean HbA_1c_ is 59.3 (SD 17.4) mmol/mol , mean systolic and diastolic BP (mm Hg) is 134.3 (SD 14.6) and 76.1 (SD 9.5) mm Hg, respectively, and mean TC is 4.1 (SD 0.98) mmol/L. Out of 2721 patients, 630 (23.15%) had achieved a tight BP target of <130/80 mm Hg and 707 patients (25.98%) had achieved a tight TC target of <3.5 mmol/L. Overall, 131 out of 2721 (4.81%) patients achieved all 3 “tight” CV risk factor targets. CV risk factor burden assessed by the prevalence of coronary heart disease, cerebrovascular disease, and peripheral vascular disease increased two-fold in those with eGFR<60 mL/min/1.73 m^2^ compared with those with eGFR≥60 mL/min/1.73 m^2^. Overall, 1076 out of 2721 (39.46%) individuals had a code for MA or proteinuria on their primary care medical record. Out of 2721 patients, 2064 (75.85%) were prescribed a nephroprotective agent, such as an angiotensin-converting enzyme inhibitor or an angiotensin receptor blocker drug, whereas 2070 patients (76.07%) were prescribed a cholesterol-lowering medication, that is, statin therapy.

**Table 1:** Biomedical characteristics of eligible study patients.

Characteristics		Control (N=1299)	Intervention (N=1422)	Total (N=2721)	*P* value
Age in years, mean (SD)		62.8 (10.1)	62.9 (9.9)	62.9 (10.0)	.63
**Age category, n (%)**					
	<30 years	4 (0.31)	3 (0.21)	7 (0.26)	
	30-44 years	67 (5.16)	63 (4.43)	130 (4.78)	
	45-65 years	623 (47.96)	712 (50.07)	1335 (49.06)	
	>65 years	605 (46.57)	644 (45.29)	1249 (45.90)	
**Gender, n (%)**					**.27**
	Male	776 (59.74)	820 (57.67)	1596 (58.65)	
	Female	523 (40.26)	602 (42.33)	1125 (41.35)	
**Ethnicity, n (%)**					**<.001**
	White	380 (38.31)	195 (23.0)	575 (31.27)	
	Black	45 (4.54)	17 (2.01)	62 (3.37)	
	Asian	516 (52.02)	620 (73.20)	1136 (61.77)	
	Mixed	14 (1.41)	6 (0.71)	20 (1.09)	
	Other	37 (3.73)	9 (1.06)	46 (2.50)	
**Smoking status, n (%)**					**.64**
	Nonsmoker	750 (57.74)	817 (57.45)	1567 (57.59)	
	Current smoker	205 (15.78)	210 (14.77)	415 (15.25)	
	Ex-smoker	344 (26.48)	395 (27.78)	739 (27.16)	
Duration of T2DM^a^ in years, mean (SD)		10.62 (7.31)	10.31 (7.14)	10.46 (7.22)	.27
HbA_1c_^b^ in mmol/mol, mean (SD)		59.8 (18.1)	58.9 (16.7)	59.3 (17.4)	.14
HbA_1c_<53 mmol/mol, n (%)		546 (42.03)	618 (43.46)	1164 (42.78)	.45
HbA_1c_<58.5 mmol/mol, n (%)		755 (58.12)	849 (59.70)	1604 (58.95)	.39
Total cholesterol in mmol/L, mean (SD)		4.1 (1.0)	4.1 (0.94)	4.1 (0.98)	.05
Total cholesterol<3.5 mmol/L, n (%)		335 (25.79)	372 (26.16)	707 (25.98)	.81
Total cholesterol<5 mmol/L, n (%)		1075 (82.76)	1211 (85.16)	2286 (84.01)	.07
Systolic BP^c^ in mm Hg, mean (SD)		134.0 (15.0)	134.5 (14.2)	134.3 (14.6)	.82
Diastolic BP in mm Hg, mean (SD)		76.7 (9.7)	75.4 (9.2)	76.1 (9.5)	<.001
BP<130/80 mm Hg, n (%)		320 (24.63)	320 (22.50)	640 (23.52)	.19
BP<140/80 mm Hg, n (%)		608 (46.81)	670 (47.12)	1278 (46.97)	.87
eGFR^d^ in mL/min, median (IQR^e^)		81.0 (62.0-90.0)	85.0 (66.0-90.0)	83.0 (64.0-90.0)	.047
**CKD^f^ Stage 3, n (%)**		260 (20.02)	276 (19.41)	536 (19.70)	.06
	Stage 3a	34 (13.08)	30 (10.87)	64 (11.94)	.38
	Stage 3b	22 (8.46)	13 (4.71)	35 (6.53)	.07
CKD Stage 4, n (%)		33 (2.54)	21 (1.48)	54 (1.98)	.05
CKD Stage 5, n (%)		20 (1.54)	13 (0.91)	33 (1.21)	.14
**Medical history, n (%)**					
	Myocardial infarction	97 (7.47)	89 (6.26)	186 (6.84)	.21
	Acute coronary syndrome	51 (3.93)	57 (4.01)	108 (3.97)	.91
	Angina	120 (9.24)	132 (9.28)	252 (9.26)	.97
	Ischemic heart disease	249 (19.17)	264 (18.57)	513 (18.85)	.68
	Transient ischemic attack	66 (55.08)	35 (2.46)	101 (3.71)	<.001
	Stroke	46 (3.54)	55 (3.87)	101 (3.71)	.65
	Peripheral vascular disease	48 (3.70)	34 (2.39)	82 (3.01)	.047
	Revascularization procedure	92 (7.08)	40 (2.81)	132 (4.85)	<.001
	Bypass graft	47 (3.62)	53 (3.73)	100 (3.68)	.88
**Number of study risk factors controlled, n (%)^g^**					**.21**
	0	336 (25.87)	337 (23.70)	673 (24.73)	
	1	579 (44.57)	680 (47.82)	1259 (46.27)	
	2	309 (23.79)	336 (23.63)	645 (23.70)	
	3	70 (5.39)	61 (4.29)	131 (4.81)	
MA^h^/proteinuria code, n (%)		410 (31.56)	666 (46.84)	1076 (39.54)	<.001

^a^T2DM: type 2 diabetes.

^b^HbA_1c_: glycated hemoglobin.

^c^BP: blood pressure.

^d^eGFR: estimated glomerular filtration rate.

^e^IQR: interquartile range.

^f^CKD: chronic kidney disease.

^g^HbA_1c_ <7.5% (58.5 mmol/mol), total cholesterol <3.5 mmol/L, BP <130/80 mm Hg.

^h^MA: microalbuminuria.

**Table 2:** Vascular burden between patient groups categorized by chronic kidney disease stages.

Disease characteristic		Patients with MA^a^ and eGFR^b^ <60			Patients with MA and eGFR>60		
		Control (N=280)	Intervention (N=258)	Total (N=538)	Control (N=1019)	Intervention (N=1164)	Total (N=2183)
**Cardiovascular disease, n (%)**							
	Myocardial infarction	33 (11.8)	29 (11.2)	62 (11.5)	63 (6.18)	58 (4.98)	121 (5.54)
	Acute coronary syndrome	19 (6.8)	16 (6.2)	35 (6.5)	32 (3.14)	41 (3.52)	73 (3.34)
	Angina	38 (13.6)	30 (11.6)	68 (12.6)	81 (7.95)	101 (8.68)	182 (8.34)
	Ischemic heart disease	78 (27.9)	75 (29.1)	153 (28.4)	169 (16.58)	186 (15.98)	355 (16.26)
	Coronary artery bypass graft	18 (6.4)	16 (6.2)	34 (6.3)	29 (2.58)	36 (3.09)	65 (2.98)
**Cerebrovascular disease, n (%)**							
	Transient ischemic attack	19 (6.8)	10 (3.9)	29 (5.4)	47 (4.61)	25 (2.15)	72 (3.30)
	Stroke	15 (5.4)	16 (6.2)	31 (5.8)	30 (2.94)	38 (3.26)	68 (3.11)
	Peripheral vascular disease	21 (7.5)	16 (6.2)	37 (6.9)	27 (2.65)	18 (1.55)	45 (2.06)
	Hypertension (BP^c^>130/80)	80 (28.6)	81 (31.4)	161 (29.9)	236 (23.16)	235 (20.19)	471 (21.58)
	Hyperlipidemia (TC^d^>4.0)	125 (44.6)	110 (42.6)	235 (43.7)	482 (47.30)	504 (43.30)	986 (45.17)

^a^MA: microalbuminuria.

^b^eGFR: estimated glomerular filtration rate.

^c^BP: blood pressure.

^d^TC: total cholesterol.

**Table 3:** Drug prescribing in eligible study individuals at baseline.

Drug	Control (N=1299), n (%)	Intervention (N=1422), n (%)	Total (N=2721), n (%)	*P* value
Long-acting insulin	225 (17.32)	268 (18.85)	493 (18.12)	.30
Short-acting insulin	92 (7.08)	123 (8.65)	215 (7.90)	.13
Metformin	926 (71.29)	971 (68.28)	1897 (69.72)	.09
Sulphonylurea	422 (32.49)	434 (30.52)	856 (31.46)	.27
Dipeptidyl peptidase-4 (DPP-4)	205 (15.78)	228 (16.03)	433 (15.91)	.86
Thiazolidinediones (TZD)	78 (6.00)	28 (1.97)	106 (3.90)	<.001
Glucagon-like peptide-1 receptor antagonist (GLP-1 RA)	56 (4.31)	39 (2.74)	95 (3.49)	.03
Sodium glucose transporter-1 inhibitor (SGLT-2i)	28 (2.16)	20 (1.41)	48 (1.76)	.14
Meglitinide	220 (16.94)	311 (21.87)	531 (19.51)	.001
Calcium channel blocker (CCB)	524 (40.34)	592 (41.63)	1116 (41.01)	.49
Diuretic	388 (29.87)	412 (28.97)	800 (29.40)	.61
Angiotensin-converting enzyme (ACE) inhibitor	666 (51.27)	713 (50.14)	1379 (50.68)	.56
Angiotensin receptor blocker (ARB)	349 (26.87)	336 (23.63)	685 (25.17)	.05
Beta blocker	376 (29.95)	407 (28.62)	783 (28.78)	.85
Alpha blocker	393 (30.25)	455 (32.00)	848 (31.17)	.23
Aspirin	393 (30.25)	455 (32.00)	848 (31.17)	.33
Clopidogrel	74 (5.70)	76 (5.34)	150 (5.51)	.69
Statin	959 (73.83)	1111 (78.13)	2070 (76.07)	.01
Fibrate	29 (2.23)	32 (2.25)	61 (2.24)	.98
Ezetimibe	26 (2.00)	37 (2.60)	63 (2.32)	.30
Warfarin	66 (5.08)	79 (5.56)	145 (5.33)	.58

## Discussion

### Overview

This paper describes the design and baseline characteristics of a pragmatic cluster RCT that will investigate the effectiveness of a multifactorial intervention. The study’s achievements thus far include the following: establishing the infrastructure for the trial, recruitment of the required number of GP practices, provision of training for HCPs at intervention practices, and baseline data extraction. Analysis of the baseline data shows poor coding levels of individuals meeting the diagnostic criteria for MA within their medical record. Subgroup analysis by eGFR highlights the increased vascular burden in patients with kidney disease.

### Principal Findings

MA is an easily available integrated marker, suggesting subclinical generalized involvement of the vascular system, predisposing individuals with T2DM to increased risk of CV disease. Evaluation of abnormal urinary albumin excretion through urinary ACR in individuals with T2DM is a specific and cost-effective method to help identify individuals who can benefit from additional intensive, targeted interventions involving tight CV risk factor control [3,28]. However, despite the plethora of evidence, UK National Audit data from 2015 to 2016 showed that only 40.2% people with T2DM were reaching all 3 treatment targets [29] (HbA_1c_<58 mmol/mol, BP<140/80 mm Hg, and TC<5 mmol/L). Although we used tighter risk factor targets in our study, which may not conform to targets set out by practicing clinicians in primary care (eg, UK QOF targets), it can be argued that the nature of a high-risk state such as T2DM with MA deserves stricter cardio-metabolic control to achieve greater CV mortality and morbidity benefits [3,28,30]. However, individualization of therapy must take precedence as emphasized in the National Institute For Health and Care Excellence (NICE) guidelines for T2DM in the United Kingdom [2] and in the new 2017 American Diabetes Association statement on standards of medical care in diabetes [31], which encourages clinicians to use a pragmatic approach and be cognizant of the “risk-benefits” while using treatments to minimize CV risk. Accordingly, although our baseline data in this high-risk population suggest “adequate to satisfactory” performance in achieving tight cardio-metabolic risk factors targets, prescribing patterns particularly relating to nephroprotective agents and statin therapy and achieving all “prescribed” care processes such as “coding for MA” could be improved.

A number of reasons may explain why a high proportion of individuals do not achieve cardio-metabolic targets in primary care, including clinical inertia, aiming only for “prescribed” QOF targets [32,33], lack of time for treating complex patients, gaps in clinician knowledge, and the need for a well-organized health care system to manage chronic conditions [34]. However, improvements in clinical care processes and targeted control of CV risk factors in high-risk individuals with T2DM and MA have recognized benefits and should be pursued without delay and with commitment, both from the perspective of the HCP and the affected individual [3,30]. Our hypothesis was that a multifaceted, multifactorial intervention including the use of an electronic “Prompt” readily available and visible to the treating HCP during “limited” consultation times would serve as an “aide-memoire” to intensify treatment targets and improve clinical outcomes.

A cluster randomized design was chosen to avoid contamination [35] which may have occurred if it was at the patient level. Furthermore, HCPs could face conflict if they were to deliver a purposeful intensive intervention only to certain individuals within their population.

The GP prompt study, to our knowledge, is the largest RCT assessing the effectiveness of a practice-level intervention to support management of a multiethnic population diagnosed with T2DM and MA. Baseline data give contemporary estimates for adherence to best practice risk factor targets and the increased vascular burden in these affected individuals.

### Limitations

An identified weakness of this trial may relate to the chosen method of data collection. To allow collection of large amounts of data and cluster randomization at the practice level, Read-coded primary care data are being collected using MIQUEST. The accuracy of the data and their usefulness as resource for study are therefore dependent on the coding practices of individual clinicians in individual practices. For example, level of coding within primary care for ethnicity has previously been found to be poor [36]. Our baseline data suggest that although recording of ethnicity data has improved, there remains significant variation between practices. This lack of complete data may limit our ability to perform secondary subgroup analysis to study interracial variations [37].

Anecdotal evidence suggests that coding quality for MA within primary care data is poor. Putative factors may include requirement for more than one sample to make a diagnosis, which may cause delays in diagnosis; infrequent testing and recall; and lack of recognition of MA as an important “CV risk marker.” Furthermore, it is likely that recommended processes of care that are performed frequently (BP, HbA_1c_, and lipid checks) may be more diligently recorded than those recommended and/or performed less frequently (eg, MA, foot examination). To account for this, we used pragmatic inclusion criteria based on international guidelines for MA [2]. We used a definition of 2 abnormal ACR values (2.5 mg/mmol for men, >3.5 mg/mmol for women) >90 days but <180 days apart [38]. Using this definition, only 1076 out of 2721 patients (39.54%) with 2 abnormal ACR values (>2.5 in males and >3.5 females) on 2 occasions >90 days and <180 days apart were coded as having MA or overt proteinuria. We were not able to exclude patients with proteinuria or urinary tract infection as per national guidance because of the methodology by which such data are coded. Despite this, prevalence rates for MA within the study population are broadly in line with other large contemporary population–based studies [39].

### Conclusions

Multifactorial-targeted interventions in individuals with T2DM and MA have shown efficacy in reducing CV events and mortality, mostly in specialist settings. However, their effectiveness, implementation, and cost-effectiveness in a primary care setting have not been adequately tested. The results of this study, including a comprehensive cost-effectiveness analysis, will inform on these issues and will be published in 2018. If the results of the GP prompt study are positive, there is a potential for “scaling up” under real-world conditions to reach a greater proportion of the eligible population. Skills, competencies, and workforce required for wider implementation would need to be assessed, and the results of this study would provide policy makers and senior decision makers with vital information to facilitate widespread adoption into CV risk reduction programs.

## Appendix

Multimedia Appendix 1 Clinical system "prompts."

Multimedia Appendix 2 Enhanced care template.

Multimedia Appendix 3 Treatment algorithm.

Multimedia Appendix 4 HCP training programme.

## Abbreviations

ACR: albumin-creatinine ratio
BP: blood pressure
CKD: chronic kidney disease
eGFR: estimated glomerular filtration rate
GP: general practitioner
HbA_1c_: glycated hemoglobin
HCP: health care professional
IQR: interquartile range
IT: information technology
MA: Microalbuminuria
MIQUEST: morbidity information query and export syntax
QOF: Quality and Outcomes Framework
RCT: randomized controlled trial
TC: total cholesterol
T2DM: type 2 diabetes
