# Supplementary material for: The General Practitioner Prompt Study to Reduce Cardiovascular and Renal Complications in Patients With Type 2 Diabetes and Renal Complications: Protocol and Baseline Characteristics for a Cluster Randomized Controlled Trial
Source: JMIR Res Protoc. 2018 Jun 8;7(6):e152. doi: 10.2196/resprot.9588 (PMC6015271; doi:10.2196/resprot.9588)

Multimedia Appendix 2.

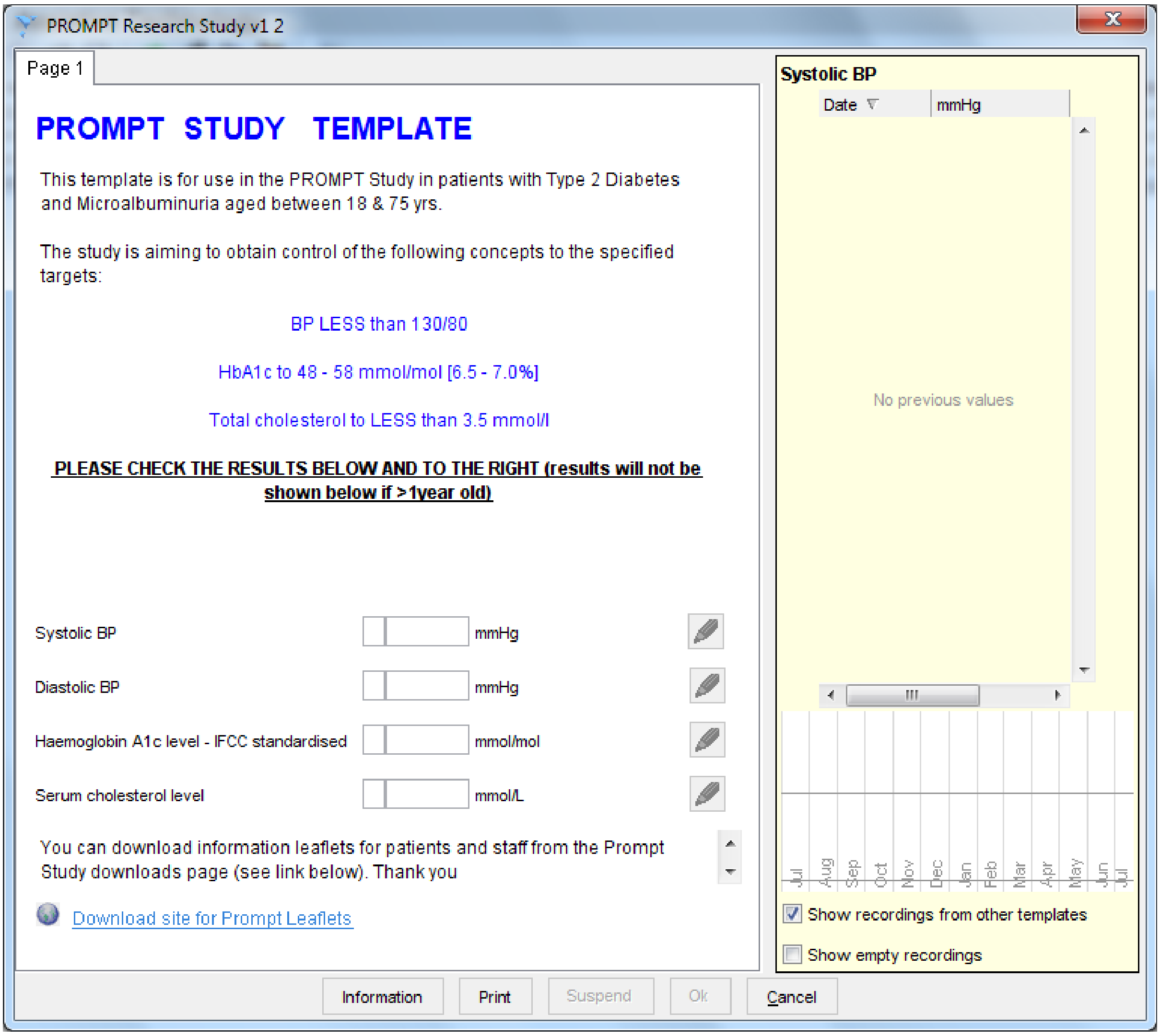

Supplement: Multimedia Appendix 2 [file resprot_v7i6e152_app2.pdf]
