# Supplementary material for: The General Practitioner Prompt Study to Reduce Cardiovascular and Renal Complications in Patients With Type 2 Diabetes and Renal Complications: Protocol and Baseline Characteristics for a Cluster Randomized Controlled Trial
Source: JMIR Res Protoc. 2018 Jun 8;7(6):e152. doi: 10.2196/resprot.9588 (PMC6015271; doi:10.2196/resprot.9588)
Supplement: Multimedia Appendix 4 [file resprot_v7i6e152_app4.pdf]

Multimedia Appendix 4.

The HCP training programme was developed by a consultant diabetologist (WAC) with the support of a diabetes specialist nurse with experience in developing and delivering training to primary care staff. The development of the curriculum was informed by focus groups with local GPs and diabetes specialist nurses. The final training curriculum was piloted at a regional meeting for healthcare professionals with a special interest in diabetes. Care was taken not to involve healthcare professionals from either of the study arms during development of the training.

The training was delivered over a three hour group session to a minimum of two HCPs from each participating practice (1 GP, 1 nurse) by the study clinician and research staff. Tcost of attending the training was reimbursed. It was expected that the staff attending the training would disseminate the information and skills they had learned to fellow colleagues at their practice and they were provided with resources to do this. The training curriculum focussed on the evidence base for adherence to tighter risk factor targets, justification for the methods used and current guidance for managing cardiovascular (CV) risk in eligible patients. Training on how to use the software prompt was also provided. Training resources were produced enclosing lecture notes, slides and examples of generic patient case histories to promote discussion amongst participants.

Ongoing support is being to intervention practices including six monthly visits to discuss issues relating to the management of eligible patients. Performance reports are provided every 3 months which show progress of eligible patients in meeting study risk factor targets in addition to practice level prescribing data for Angiotensin converting enzyme inhibitors (ACEi) or Angiotensin receptor blockers (ARBs), statins and antiplatelet agents amongst eligible patients.
